# Aiding Reflective Navigation in a Dynamic Information Landscape: A Challenge for Educational Psychology

**DOI:** 10.3389/fpsyg.2022.881539

**Published:** 2022-05-02

**Authors:** Katarzyna Bobrowicz, Areum Han, Jennifer Hausen, Samuel Greiff

**Affiliations:** Computer-Based Assessment Group, Department of Behavioural and Cognitive Sciences, University of Luxembourg, Esch-sur-Alzette, Luxembourg

**Keywords:** open access, COVID-19, 21st century skills, health literacy, critical literacy, statistical literacy, metacognition, rational thinking

## Abstract

Open access to information is now a universal phenomenon thanks to rapid technological developments across the globe. This open and universal access to information is a key value of democratic societies because, in principle, it supports well-informed decision-making on individual, local, and global matters. In practice, however, without appropriate readiness for navigation in a dynamic information landscape, such access to information can become a threat to public health, safety, and economy, as the COVID-19 pandemic has shown. In the past, this readiness was often conceptualized in terms of adequate literacy levels, but the contemporarily observed highest-ever literacy levels have not immunized our societies against the risks of misinformation. Therefore, in this Perspective, we argue that democratization of access to information endows citizens with new responsibilities, and second, these responsibilities demand readiness that cannot be reduced to mere literacy levels. In fact, this readiness builds on individual adequate literacy skills, but also requires rational thinking and awareness of own information processing. We gather evidence from developmental, educational, and cognitive psychology to show how these aspects of readiness could be improved through education interventions, and how they may be related to healthy work-home balance and self-efficacy. All these components of education are critical to responsible global citizenship and will determine the future direction of our societies.

## Introduction

We live in the age of nearly universal access to information. With decentralized news outlets, growing access to open science, and worldwide social media coverage, individuals can be more broadly and diversely informed than ever before. Open access to information is a key value of modern democratic societies, as only thoroughly informed citizens can participate in society and make informed decisions about the directions in which they wish their society to evolve. It seems, however, that despite open and multisource access to information, individuals fail to make thoroughly informed choices at both societal and individual levels. In this Perspective, we aim to examine why such failures may happen and how they could be remedied in education.

**FIGURE 1:**
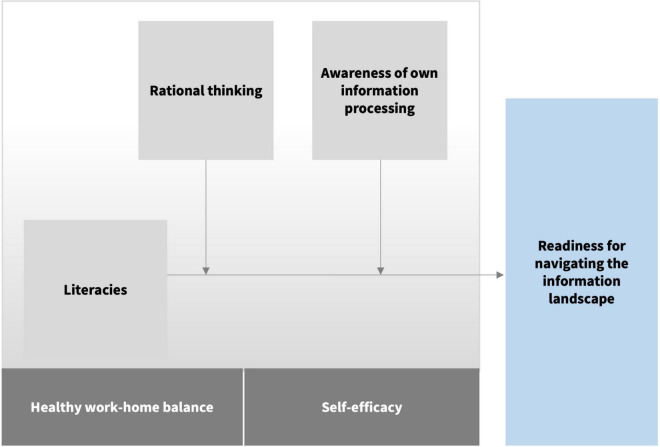
The proposed model of the relationship between literacies and readiness for navigating the information landscape, moderated by rational thinking and awareness of own information processing. Here, literacies are defined as “competencies and dispositions that support individual comprehension and use of information in daily activities”; rational thinking as “reflective, effortful processing of information”; and awareness of own information processing as “using psychological knowledge to take stock of own mental operations”. Healthy work-home balance and self-efficacy are also components of the educational sphere that may contribute to readiness for navigating the information landscape.

The ongoing COVID-19 crisis exposed, and perhaps augmented, society-wide difficulties with critical processing of dynamically changing information. This may have impeded many important outcomes such as sufficiently high vaccination rates, which, in turn, impose health, social, and economic losses on entire societies. Misinformation and other environmental factors outside of an individual’s control have certainly contributed to such losses (Okuhara et al., 2020; Engledowl and Weiland, 2021; Montagni I. et al., 2021). However, in this Perspective, we focus on the lack of individual readiness for navigating the dynamic information landscape, which may exacerbate poor decision-making under the pressing conditions of the prevailing global crisis. As educational psychologists, we suggest that equipping citizens with such readiness is a pressing challenge for contemporary education that needs to be targeted at all stages of individual development across the lifespan. We are certainly not alone in this view, as prolific literacy research has shown for several decades (e.g., Cervetti et al., 2006; Leu et al., 2017). Literacies are essential competencies, skills and dispositions that support individual comprehension and use of “information in daily activities to achieve goals, develop knowledge and potential” (cf. Organization Economic Cooperation Development [OECD], 2000, p. x; Leu et al., 2017). Since improving literacies is only part of the solution, we sketch out a more complex and comprehensive picture of the challenge ahead by coupling literacy research with relevant research on rational thinking and cognition. We argue that fostering responsible citizenship demands, at the very least, literacies, rational thinking (i.e., reflective, effortful processing of information), and awareness of our own information processing (i.e., using psychological knowledge to take stock of own mental operations; Figure 1). Throughout the paper, we recount evidence-based solutions developed within each of these research fields to offer concrete suggestions for education stakeholders.

## Open Access, Literacies, and Rational Thinking

### Open Access Endows the Citizen With New Responsibilities

Openness and Participation are critical to democratic citizenship (Knight Commission Report, 2009; Şimşek and Şimşek, 2013). Openness implies that everybody can reach the information that supports civic and personal decisions, which serves to facilitate Participation, that is, joining and using information systems to solve civic and personal issues (Knight Commission Report, 2009). As open and multisource access to information secures these values, it cannot be realistically controlled by democratic governments (Eysenbach, 2008). This emancipation from top-down control forces individual citizens to assume responsibility for their own navigation in the dynamic information landscape (Weiland, 2017). Citizens need to learn how to identify and critically process relevant information (Eysenbach, 2008), and how to position themselves in relation to a myriad of views, values, and ideas (Weiland, 2017). The COVID-19 crisis is one example that clearly showed that citizens were not ready for such navigation.

Soon before the onset of the crisis, vaccine hesitancy, a delay in acceptance, or a refusal of vaccine despite its availability (MacDonald and SAGE Working Group on Vaccine Hesitancy, 2015; Montagni I. et al., 2021), was listed among the 10 critical global threats by the World Health Organization [WHO] (2019), Okuhara et al. (2020). Hesitant attitudes toward vaccines became increasingly studied during the COVID-19 crisis, showing that they were associated with poor health literacy (Montagni I. et al., 2021; Turhan et al., 2021; in particular, digital health literacy, Patil et al., 2021) and a mismatch between affect-laden anti-vaccination messages and statistics-based governmental communication (Okuhara et al., 2020). In the cognitively and emotionally challenging “infodemic,” understood as an overabundance of information on virus-related matters (Duplaga et al., 2019; World Health Organization [WHO], 2020; Patil et al., 2021), the citizens were left to their own devices. Despite higher-than-ever literacy rates, the public was poorly prepared for universal access to information and failed to assume the responsibilities that come with such access. Paired with poor readiness for selecting, evaluating, and processing information, universal access to information became a threat, not an aid.

### Literacies Are Key but Insufficient

Universal access to information is here to stay but using it to individual and societal advantage rather than disadvantage demands better readiness, and it is better to foster it late than never. The notion that the age of access demands specific skills, and that educational systems across the world should support individuals in honing these skills, is not new. For over two decades now, educational psychologists have investigated a myriad of literacy skills (i.e., literacies) that support individual navigation in the dynamically changing information landscape (Cervetti et al., 2006; Şimşek and Şimşek, 2013; Leu et al., 2017). Literacy became a popular buzzword in educational psychology and an umbrella term for multiple, multimodal and multifaceted skills (Cervetti et al., 2006). Literacy for twenty-first century citizenship is digital, complex, and dynamic, but it builds on traditional literacies, such as reading, writing and comprehension (Leu et al., 2017). These traditional literacies need to be applied in the digital world, in which information is no longer provided in a single modality (e.g., audio only), a single flow (e.g., without simultaneous advertising), or by a limited group of individuals sharing similar values, views, and ideas. The line between the addresser and the addressee is fuzzy, as each member of the online community may smoothly move on the continuum between these roles. Therefore, twenty-first century literacies demand not only technical readiness for the modern technologies, but, critically, control over one’s own information seeking and processing, awareness of the complexity of the social world (Cervetti et al., 2006; Leu et al., 2017), and a balance between emotional engagement and emotional distance toward arguments presented by others (Gee, 2012; Olin-Scheller and Tengberg, 2017).

Technical readiness is typically a minor challenge for “digital natives” (Prensky, 2001; Valenza, 2006; Harris, 2008) or “insiders” (Lankshear and Knobel, 2006; Wimmer and Draper, 2019), who are children and adolescents raised amid rapidly evolving technologies from their birth (Neumann, 2016). However, evidence from educational and developmental psychology showed that digital natives, despite technical readiness, lack critical literacy and struggle with seeking, selecting, and evaluating information, and are not by default skilled or critical consumers of information (Valenza, 2006; Harris, 2008; Wineburg et al., 2016). Digital natives typically prioritize most accessible information sources and effortless processing (Shenton and Dixon, 2004; van Deursen et al., 2014; Loh and Kanai, 2015), and show age-specific difficulties in deploying attention to relevant information. Younger children (at 8–10 years) struggle with inhibiting irrelevant yet salient and engaging information (Eastin et al., 2006) and typically do not question its accuracy (Hirsh, 1999); older children lack sufficient knowledge base and analytical skills to contextualize and analyze the information, and often prioritize the form of the information over its content (Watson, 1998; Agosto, 2002; Sundar, 2008). This suggests that critical literacy learning should be adjusted to children’s cognitive developmental stage (Eastin, 2008) and fostered in education (Bernstein, 2000; Gee, 2012; Leu et al., 2017; Olin-Scheller and Tengberg, 2017) because it is central to the new responsibilities that follow from universal access to information. Critical literacy, “the ability to argue from evidence, values and different perspectives (Skolverket., 2011) and to go beyond the individual and personal, and relate knowledge to more general and abstract notions (Bernstein, 2000; Gee, 2012),” as defined by Olin-Scheller and Tengberg (2017, p. 428), must be paired with age-appropriate statistical literacy and scientific literacy to prepare the prospective citizen for the influx of complex, heavily data-based information (Gal, 2002; Organization Economic Cooperation Development [OECD], 2004; Engel, 2017, 2019; Podkul et al., 2020; Engledowl and Weiland, 2021). To this end, in educational settings, complex information should not be simplified, but rather turned into manageable chunks and presented on multiple occasions (Spiro et al., 2003). Comparing poorly presented information, e.g., misleading data visualizations with well-executed visualizations provided by such tools as Engel (2019), Gapminder (2022) should be also a regular part of classroom activities (Engledowl and Weiland, 2021).

Critical literacy relies on cognitive skills, but it cannot be achieved without Students’ emotional investment. Educational psychologists showed that students need to learn how to self-regulate their own investment by striking a balance between emotional engagement and emotional distance toward their own and others’ arguments when discussing social issues (Gee, 2012; Olin-Scheller and Tengberg, 2017). Inhibiting own emotional attitudes is a prerequisite when evaluating own and others’ arguments and can be effectively trained in the classroom context, by, for instance, drawing Students’ attention to the affect-laden but irrelevant arguments that they present in response to a controversial issue (Olin-Scheller and Tengberg, 2017). This requires metacognition, understood as active monitoring of own thoughts and emotions, and switching between own and others’ perspectives (Flavell, 1976). Although this ability develops early (Marulis et al., 2016), it becomes an educational priority only in adolescence (Eysenbach, 2008; Olin-Scheller and Tengberg, 2017). In adolescence, citizens become increasingly autonomous, and drift away from reliance on traditional authority figures (i.e., intermediaries; Eysenbach, 2008) toward support from peers and less authoritative sources of information (i.e., apomediaries; Eysenbach, 2008). Individual autonomy continues to develop in adulthood, and is typically associated with gains in cognitive ability, literacies, and self-efficacy. Low capacity and/or insufficient belief in one’s own capacity can push even adult citizens into reliance on traditional authority figures and uncritical, unreflective processing of information (Eysenbach, 2008). Therefore, critical literacy is only a bare minimum for individual readiness during global crises.

### Literate Citizens Are Not Rational Thinkers

Navigating the information landscape demands critical literacy, paired with other relevant literacies, such as media literacy, health literacy, and statistical literacy. To date, various interventions have aimed to boost relevant literacies in individuals, perhaps stemming from a belief that a sufficiently literate individual will be able to scan, select, and evaluate the information landscape to make thoroughly informed choices. Empirical research findings on human rationality, however, suggest otherwise, showing that literacies are fundamental but not sufficient for individual readiness. Several models of human cognition showed that information processing roughly follows two separate routes: either a peripheral, fast route with mental shortcuts and simple rules (i.e., heuristics) or a central, cognitively effortful route with a reflective, metacognitive outlook on the available information (Sundar, 2008; Stanovich, 2009, 2018). Having access to these two processing routes (or systems, Stanovich, 2009) is highly adaptive, but overreliance on the heuristic route can lead to multiple cognitive biases (Sundar, 2008; Stanovich, 2009). Therefore, people who are sufficiently literate in terms of health and statistical knowledge may nevertheless suffer such cognitive biases (Stanovich, 2018) and make irrational choices as to whether, for example, to vaccinate themselves and/or their children against COVID-19 due to the anti-vaccination messages. The messages are typically communicated through affect-laden, salient imagery focused on vaccine toxicity and its side effects and consequently tend to trigger fast, heuristic processing (Okuhara et al., 2020). Even individuals that reflectively weigh the benefits and risks of the vaccine against the risks of COVID-19 infection may struggle with overriding the worry caused by the heuristic processing of such information (Okuhara et al., 2020).

As the above example showed, cognitive psychologists have repeatedly shown that individuals are prone to the same biases across social strata regardless of their literacy levels, but they may be able to override these biases with sufficient awareness and training (Stanovich, 2018). Instead of resorting to fast, automatic processing that prioritizes ease and speed over rationality, individuals should be able to intentionally choose slow, reflective processing of the information landscape. Educating citizens on the multiple heuristics triggered by salient but irrelevant aspects of communication (Sundar, 2008) may be a good introduction to rational thinking. To some extent, training of rational thinking overlaps with literacy training, as they also involve technical components of statistical and scientific literacy (Stanovich, 2009). Such technical skills involve, among others, learning probability theory, switching between absolute and relative representations of data (e.g., 1,000 individuals vs. 25% of the tested sample vs. 1 in 4 tested people got infected) and overriding an automatic tendency to overestimate absolute numbers compared to percentages (Yamagishi, 1997; Stanovich, 2009). Thus far, however, it seems that citizens, lacking systematic training on this matter, have not had a chance to build an awareness of their own cognitive functioning. Without such chances at a society-wide level, many citizens will not be able to take control over their own cognitive functioning, and, thereby, will not achieve readiness for universal access to information. Their information processing will be governed externally, by those who manipulate vividness, accessibility, and salience of information (Stanovich, 2009), such as advertising companies in the modern market-based society. Thus far, the lack of awareness on own information processing had not threatened the economy, health, and survival, and had been overlooked in education. Now that it is generating considerable economic losses for the local and global communities, it will finally find its way to society-wide educational practices. We thus propose that developing a habit of taking stock of one’s mental operations should become more prominent in the education of children, adolescents, and adults.

Better understanding of one’s own information processing is a goal that demands concrete, structured action, with the concept of (ir)relevance of information at its core (Heine, 2006; Eysenbach, 2008; Hobbs and Jensen, 2009; Román et al., 2009; Olin-Scheller and Tengberg, 2017; Bajo et al., 2021). Identifying relevant information is key for critical and rational thinking, since changes of salient, irrelevant information can significantly impact human decision-making (i.e., framing effect; Stanovich, 2009). This skill has been championed by several scholars in recent years, pointing toward conditions in which students can efficiently take responsibility for handling access to information (Lankes, 2008; Weingarten, 2008; Rapp and McCrudden, 2018). Providing students with information of varying relevance and credibility, both in the classroom and in complex real-world contexts, gives them the best opportunity to determine on their own what matters regarding the information flow (Lankes, 2008; Weingarten, 2008; Rapp and McCrudden, 2018). Furthermore, delegating the responsibility for setting up own sub-goals, and selecting information without micromanaging instruction leads to more accurate relevance judgments (Heine, 2006). One way to achieve such tasks may be to construct self-regulated and metacognitive learning environments where students can figure out their own information processing with the help of technologies in learning analytics (e.g., Winne et al., 2019) and cooperation with interdisciplinary professionals. Completing such tasks may further build Students’ confidence in their own information processing abilities and steer them toward autonomous, critical thinking.

## Discussion

Over two centuries ago, a French mathematician argued during the French Revolution that citizens need *savoir liberateur*, knowledge built on information raising their awareness of the state of society (De Condorcet, 1994; Engel, 2019). The concept of a well-informed citizen is not new (e.g., Schutz, 1964; Strassheim, 2018), but it is, perhaps more than ever, pivotal for contemporary societies. The COVID-19 crisis showed that offering citizens universal access to information is insufficient, and, under some circumstances, detrimental to the common good. Global citizenship demands multiple literacies, rational thinking, and awareness of own information processing.^1^ Although broad views of scientific literacy (Choi et al., 2011), critical statistical literacy (Engledowl and Weiland, 2021), and civic statistics (Engel, 2019) have incorporated metacognitive aspects of such literacies, neither rational thinking nor awareness of own information processing have been incorporated into twenty-first century skills (Dede, 2009). This demands better integration into curricula for school-aged children and adolescents, and informational interventions for adults.

Improving cognitive abilities or an overview of such abilities will not suffice when facing the challenges of the twenty-first century. Therefore, beyond the curricula and interventions at a broader level, we posit that readiness, relevance, and responsibility are important keywords for education in the 2020s, the decade of crucial decision-making that will shape the life of every individual in the global community. Given that readiness for responsible citizenship relies on rational thinking, and that overworked or emotionally burdened individuals may be more likely to engage in fast, heuristic processing rather than slow, effortful processing of continuously incoming information (cf. Gillard et al., 2009; Spiliopoulos et al., 2018), we suggest that children, adolescents, and adults are encouraged to replace unhealthy, workaholic habits with healthy work-home balance (e.g., Deloitte Consulting, 2010; Bannai and Tamakoshi, 2014; Anxo et al., 2017; Anxo and Karlsson, 2019). Therefore, unhealthy habits instilled in school-age children and adolescents through a tremendous amount of schoolwork should be limited to foster intentional deployment of cognitive resources from an early age. To our knowledge, this has not been investigated in the past, but future experimental studies could, for instance, longitudinally track adults on a spectrum of such habits and their likelihood of using heuristic vs. reflective processing of information. Scholars from educational, developmental, and cognitive psychology need to assume this broad outlook on individual cognition, socioemotional skills and socioeconomic pressures to collaboratively and holistically foster responsible citizenship across all ages, from kindergartners to adults (see, e.g., Ellemers, 2021).

Literacy, rational thinking and work-life balance are vital parts of responsible citizenship, but it also requires sufficient self-efficacy that shields individuals against overreliance on authority figures (Eysenbach, 2008). Perceived lack of own impact on the society and non-meaningful, poorly paid jobs or unpaid internships cannot build such self-efficacy in the young citizens. Therefore, we urge adults to include young citizens in important decision-making, and to offer them meaningful, well-paid opportunities to contribute to society and to shape the world of tomorrow. Arguably, young citizens are leaving the educational system with better readiness for the contemporary digital world than older generations (Wimmer and Draper, 2019) and have high stakes in responsible, well-informed decision-making. However, without secure pay and a meaningful job, young citizens will not be able to achieve financial and intellectual autonomy critical to their participation in society.

Universal access to information is a double-edged sword. It gives all citizens a chance to shape their societies, but backfires without their readiness for responsible, well-reasoned choices. In this Perspective, based on evidence from educational, cognitive, and developmental psychology, we showed examples of concrete actions that can be taken by education stakeholders to foster citizen readiness. Since the twenty-first century globalization will certainly continue to scale up poor individual choices into international emergencies, citizen readiness, an essential remedy to such emergencies, should become a top priority for educational psychology.

## Data Availability Statement

The original contributions presented in the study are included in the article/supplementary material, further inquiries can be directed to the corresponding author/s.

## Author Contributions

KB: conceptualization, investigation, writing—original draft, writing—review and editing, and artwork. AH: conceptualization, investigation, writing—review and editing, and artwork. JH and SG: conceptualization, investigation, and writing—review and editing. All authors contributed to the article and approved the submitted version.

## Conflict of Interest

The authors declare that the research was conducted in the absence of any commercial or financial relationships that could be construed as a potential conflict of interest.

## Publisher’s Note

All claims expressed in this article are solely those of the authors and do not necessarily represent those of their affiliated organizations, or those of the publisher, the editors and the reviewers. Any product that may be evaluated in this article, or claim that may be made by its manufacturer, is not guaranteed or endorsed by the publisher.

## Footnotes

1 Global citizenship, in fact, demands more than education of individuals. In this Perspective, we have focused on such education, but we acknowledge that education of individuals must be coupled with broader environmental and societal changes. On the environmental level, for instance, citizens need external support in rational decision-making, e.g., by offering them prospectively, not temporarily, beneficial default choices (like pension funds or savings plans; Stanovich, 2009). On the societal level, for example, open and universal access to evidence-based information should be facilitated world-wide in order to alleviate post-colonial inequalities in such access (Santoro Lamelas and Belli, 2018).
