# Corrigendum: Aiding Reflective Navigation in a Dynamic Information Landscape: A Challenge for Educational Psychology

**DOI:** 10.3389/fpsyg.2022.939190

**Published:** 2022-06-09

**Authors:** Katarzyna Bobrowicz, Areum Han, Jennifer Hausen, Samuel Greiff

**Affiliations:** Computer-Based Assessment Group, Department of Behavioural and Cognitive Sciences, University of Luxembourg, Esch-sur-Alzette, Luxembourg

**Keywords:** open access, COVID-19, 21st century skills, health literacy, critical literacy, statistical literacy, metacognition, rational thinking

In the original article, there was an error in Introduction, Paragraph 1. “We in the age of nearly universal access to information. With decentralized news live outlets” should read “We live in the age of nearly universal access to information. With decentralized news outlets”. A correction has been made to Introduction, Paragraph 1:

We live in the age of nearly universal access to information. With decentralized news outlets, growing access to open science, and worldwide social media coverage, individuals can be more broadly and diversely informed than ever before. Open access to information is a key value of modern democratic societies, as only thoroughly informed citizens can participate in society and make informed decisions about the directions in which they wish their society to evolve. It seems, however, that despite open and multisource access to information, individuals fail to make thoroughly informed choices at both societal and individual levels. In this Perspective, we aim to examine why such failures may happen and how they could be remedied in education.

The authors apologize for this error and state that this does not change the scientific conclusions of the article in any way. The original article has been updated.

## Publisher's Note

All claims expressed in this article are solely those of the authors and do not necessarily represent those of their affiliated organizations, or those of the publisher, the editors and the reviewers. Any product that may be evaluated in this article, or claim that may be made by its manufacturer, is not guaranteed or endorsed by the publisher.

